# Aging and Olfactory Training: A Scoping Review

**DOI:** 10.1093/geroni/igae044

**Published:** 2024-05-30

**Authors:** Megan Loughnane, Victoria Tischler, Reem Khalid Saifeldeen, Emily Kontaris

**Affiliations:** School of Psychology, University of Surrey, Guildford, UK; School of Psychology, University of Surrey, Guildford, UK; School of Psychology, University of Surrey, Guildford, UK; Health and Well-Being Centre of Excellence, Givaudan UK Limited, Ashford, UK

**Keywords:** Cognition, Olfaction, Smell training, Well-being

## Abstract

**Background and Objectives:**

Decreased olfactory function commonly occurs alongside the aging process. Research suggests olfactory training (OT) has the potential to improve olfactory and cognitive function in individuals with and without olfactory dysfunction. The degree to which these benefits extend into older age and among those with cognitive impairment (i.e., people with dementia and mild cognitive impairment) is less clear. The purpose of the current review was to investigate the extent to which OT affects olfactory function, cognition, and well-being among older people.

**Research Design and Methods:**

A scoping review of the literature was conducted in PubMed, Embase, EbscoHost, and SCOPUS. Articles were considered eligible for original research studies with human populations, included adults aged 55 and older, performed any type of OT, and included a form of olfactory testing. The data from the included studies were synthesized and presented narratively.

**Results:**

A total of 23 studies were included. The results suggest that OT provides multiple benefits to older adults, including those with cognitive impairment. Particularly, OT was associated with measurable changes in olfactory function, improved cognitive function, specifically semantic verbal fluency and working memory, reduced depressive symptoms, and protection from cognitive decline.

**Discussion and Implications:**

The findings suggest that benefits from OT extend beyond changes in olfactory function and include improved cognitive function, amelioration of depressive symptoms, and protection from cognitive decline. Future research is needed across specific participant groups, including those with differentiated types of dementia, to investigate the olfactory and cognitive benefits of OT.

**Translational Significance:** Olfactory dysfunction is associated with increased age and neurodegenerative diseases. Olfactory training (OT) has shown benefits for improving olfaction and other cognitive domains including memory and well-being. This review examined the benefits of OT among an adult population, including those with cognitive impairment. OT was associated with measurable changes in olfactory function, improved cognitive function, specifically semantic verbal fluency and working memory, reduced depressive symptoms, and protection from cognitive decline. OT programs have the capability to be adaptive, available at low cost, and have the potential for real benefit for adults with and without cognitive impairment.

## Background and Objectives

### Olfaction and Normal Aging

Decreased olfactory function has been associated with aging by a number of studies, which identified increased age as corresponding to increased smell dysfunction (e.g., Choi et al., 2018; Hedner et al., 2010; Hummel et al., 1997; Kern et al., 2014; Larsson et al., 2000, 2009; Schubert et al., 2017; Stuck et al., 2006). In fact, most people over the age of 65 have some form of olfactory dysfunction. This ranges from mild loss, or hyposmia, to anosmia, which is a total loss of smell (Doty, 2017; Doty & Kamath, 2014). It is estimated that among the general population 3.8%–5.8% of individuals have anosmia, with prevalence rising to 13.9% in those aged 65 and older. Anosmia prevalence rises steeply among older people where up to 50% of those aged 65–80 years old have anosmia, rising to 80% for individuals over the age of 80 (Brämerson et al., 2007; Hüttenbrink et al., 2013; Karpa et al., 2010; Schubert et al., 2012). Despite the prevalence of olfactory dysfunction, it often goes unnoticed as it is rarely tested for by clinicians and fewer than 25% of those with olfactory dysfunction are aware of their deficit until tested (Doty & Kamath, 2014).

The process of olfaction relies on a combination of sensory and cognitive processes. The acuity of olfactory sensory abilities is the primary driver for odor detection, perceived odor intensity, and quality discrimination (Olofsson et al., 2021). Impaired odor identification and discrimination ability among older adults have been reported by multiple groups (Hedner et al., 2010; Kern et al., 2014; Larsson et al., 2000, 2004; Oleszkiewicz et al., 2019; Olofsson et al., 2010; Pinto et al., 2014; Schlosser et al., 2020; Schubert et al., 2011; Wilson et al., 2011). These deficits relate in part to the increased cognitive demands of the tasks as compared with threshold (which is the lowest concentration at which an odor can be detected) and intensity (the perceived strength of the odor) measurements, which are believed to reflect the function of the peripheral structures of the olfactory system (Hedner et al., 2010; Makowska et al., 2011). Although generally involving a multiple-choice design, odor identification requires detection sensitivity, quality discrimination in combination with cognitive, often multimodal abilities, including recognition memory, semantic knowledge, word retrieval, and odor–visual integration (Hedner et al., 2010; Larsson et al., 2005; Olofsson & Gottfried, 2015; Olofsson et al., 2021). While there are age-related changes across the different types of olfactory functions, the nature of these changes, and how they may vary across different types of odors, remains uncertain.

### Cognition and Neurodegenerative Disease

Olfactory dysfunction is closely associated with age-related neurodegenerative diseases and changes in olfactory function may even serve as an early biomarker for neurodegeneration (Dan et al., 2021; Doty, 2017). Persons with dementia and those with Alzheimer’s disease (AD) tend to have exacerbated or elevated olfactory deficits (Devanand, 2018; Rahayel et al., 2012; Sun et al., 2012). AD pathology includes olfactory brain regions, and performance on standardized olfactory assessments has correlated with dementia-related biomarkers (Braak & Braak, 1997; Reijs et al., 2017). Furthermore, olfactory functions tend to correlate with the cognitive abilities that decline in AD, and the olfactory deficits among persons living with AD are more pronounced in the cognitively demanding olfactory tests such as odor identification, which relies on memory and semantic activation (Olofsson et al., 2021; Rahayel et al., 2012).

Olfactory stimulation can help alleviate behavioral and psychological symptoms associated with dementia, such as aggression, agitation, hallucinations, and irritability (Fujii et al., 2008; Holmes et al., 2002). Other studies have demonstrated that olfactory training (OT) was beneficial not only to the olfactory status of persons with dementia but also reduced depressive symptoms (Cha et al., 2022; Wegener et al., 2018). While some studies report a positive effect of OT, others have reported no effect (Gray & Clair, 2002), and there is still no consensus regarding OT and those with neurodegenerative conditions.

### Olfaction, Well-Being, and Health

Olfactory dysfunction may be an indicator for well-being and psychological conditions. A decreased ability to smell has been associated with well-being factors such as increased anxiety, depression, reduced quality of life (QoL), and negative emotions (Croy et al., 2014; Eliyan et al., 2021; Kamath et al., 2023). In older adults, higher rates of depression and lower QoL have been associated with olfactory disorders, even after controlling for cognitive decline (Gopinath et al., 2012; Seo et al., 2009).

Olfaction plays a large role in eating as the perceived taste of a food is deeply influenced by the olfactory experience. Olfactory dysfunction can therefore have serious consequences on the experiences and enjoyment of food perception leading to negative outcomes such as malnutrition, changes in weight, food poisoning, and exposure to dangerous chemicals (Ferris & Duffy, 1989; Glezer et al., 2021; Tafalla, 2013).

Patient reports of the impact of smell loss have captured the negative emotions and social consequences associated with their condition. Individuals with olfactory dysfunction may have challenges in perceiving and detecting their own body odor, which can lead to embarrassment, alienation, anger, and increased feelings of isolation (Keller & Malaspina, 2013). With further respect to personal hygiene, two studies, respectively, reported that 19% and 36% of patients decreased awareness of personal hygiene was the most negative part of their olfactory disorder experience (Blomqvist et al., 2004; Nordin et al., 2011).

Not only has diminished olfactory function been proposed as an indicator for dementia and other neurodegenerative diseases, but it also serves as a marker of mortality among older adults. For adults with impaired odor identification, skewing toward the anosmic range, this deficit is independently associated with increased rates of mortality over 4–5 years (Devanand et al., 2015; Liu et al., 2019; Pinto et al., 2014). Pinto and colleagues (2014) investigated mortality and olfaction, and reported that adults with impaired olfactory identification had a 46% higher cumulative risk of death at Year 10 compared with adults who had good or normal olfactory identification. After they adjusted for confounding factors (e.g., smoking, alcohol abuse, frailty, cognitive health, etc.), older adults with anosmia had 3.37 times the odds of mortality compared to those with normal olfactory function, and this was the highest known independent leading cause of death analyzed. Despite these figures, little is known about the specific nature of olfactory loss that occurs alongside the aging process, and even less about the impact that OT could have to potentially assuage olfactory deficits and also associated cognitive and well-being factors.

### Olfactory Testing

Most studies on olfactory testing and evaluation of olfactory function refer to the domains of threshold detection, odor discrimination, and odor identification. Although a variety of assays have been developed (Hugh et al., 2015; Lawton et al., 2016), among European studies, the Sniffin’ Sticks test (Burghdart, Wedel, Germany) is a commonly-used method to measure olfactory function. The Sniffin’ Sticks test comprises three subtests and provides four scores of olfactory function: odor threshold (T), discrimination (D), identification (I), and TDI global olfactory score. The test involves the smelling of the Sniffin’ Stick (based on felt tip pen design), which contains 4 ml of odorant dissolved in propylene glycol. Threshold is assessed using the staircase method as described in detail by Kern and colleagues (2015). The staircase protocol is administered until seven reversals are observed and the dilutions presented at the last four reversals are averaged to provide the threshold score. The threshold test is time- and labor-intensive, and relies on strict constraints of the environment, making it difficult to administer and impractical in most field settings (Kern et al., 2015; Rumeau et al., 2016).

The discrimination test requires 16 triplet Sniffin’ Sticks, where two of the Sniffin’ Sticks contain the same odor and the third has a different/target odor. In the task, the participant is instructed to identify which Sniffin’ Stick has the different/target odor from the other two Sniffin’ Sticks. The identification test also requires 16 Sniffin’ Sticks, each presented, and the participant is instructed to make a forced choice from a list of four written options to identify the odorant in the Sniffin’ Stick.

Finally, the TDI score corresponds to a global olfactory score. This is calculated by summing the threshold, discrimination, and identification scores. The TDI score allows for classification of olfactory function, such as anosmia, normosmia, and hyposmia.

The University of Pennsylvania Smell Identification Test (UPSIT; Doty et al., 1984) is another widely used assessment of olfactory function. The UPSIT is a 40-item smell identification test that consists of four sets of 10 microencapsulated odors that are scratched and sniffed. Consistent with the procedure of the Sniffin’ Sticks, the respondent is asked to smell the odor and make a forced-choice response from a list of four options.

Outside of measure of odor threshold, discrimination, and identification, odor intensity ratings provide a rating of the perceived intensity of an odor on a scale ranging from very weak to very strong (Doty, 2019). Odor intensity rating scales are typically presented in a Likert-type scale with number-based ratings, for example, 0–10 where 0 is not intense and 10 is very intense (e.g., Oleszkiewicz et al., 2021) or oral descriptors. While the olfactory tests described here only highlight a few of the most used olfactory tests, the results of these psychophysical tests are correlated with each other and are believed to measure the same underlying physiologic processes (Doty et al., 1994).

### Olfactory Training

The impact of olfactory decline is multifaceted and consequential especially for older people. This decline may not be inevitable nor irreversible. The extent to which the olfactory and associated cognitive regions remain plastic is uncertain among older adults. It is widely agreed that the olfactory epithelium and other components of the olfactory pathway have regeneration capabilities (Lazarini & Lledo, 2011; Schwob et al., 1999; Youngentob & Kent, 1995), but the extent to which these extend into advanced adulthood is less clear (Olivia et al., 2022). Research findings demonstrate that OT can produce benefits to patients experiencing olfactory loss from varying etiologies (e.g., postinfectious, post-traumatic, idiopathic loss, or age-related loss; Damm et al., 2014; Hummel at al., 2009; Wegener et al., 2018).

The classical olfactory training (COT) approach was first described by Hummel and colleagues (2009) in the treatment of patients with severe olfactory loss. COT involves smelling a set of four odorants representative of different odor categories (rose [phenyl ethyl alcohol {PEA}], eucalyptus [eucalyptol], lemon [citronellal], and cloves [eugenol]), twice a day (morning and evening) for a period of 12 weeks. During the training period, patients were asked to smell the odors for 10 s each, focusing their attention on the training, and to keep a smell diary where they self-rated their olfactory function once a week. Hummel and colleagues (2009) reported that COT resulted in improved olfactory function, and for nearly 30% of participants, the improvement was clinically significant. Other studies have replicated the olfactory improvements of COT, with some reporting benefit from OT with varying treatment periods, and odor characteristics (Chen et al., 2022; Geißler et al., 2014; Wegener et al., 2018).

The benefits of OT are promising, especially given the potential for this to extend beyond olfaction, into cognition and well-being. The extent to which OT has an impact on olfactory function, cognition, and well-being among the older population has yet to be fully characterized. Many questions persist not only in the methodology of OT itself, but the potential benefits it may have for not only normally aging adults but those experiencing olfactory dysfunction and cognitive impairment.

This scoping review addresses the following research questions: (1) what assessment and methodologies are used for OT among adults aged 55 and older? (2) what outcomes are associated with OT, in terms of olfaction, cognition, and well-being? (3) what are the implications for future research involving OT among older people?

## Research Design and Methods

### Scoping Review

#### Design

**Figure 1.**
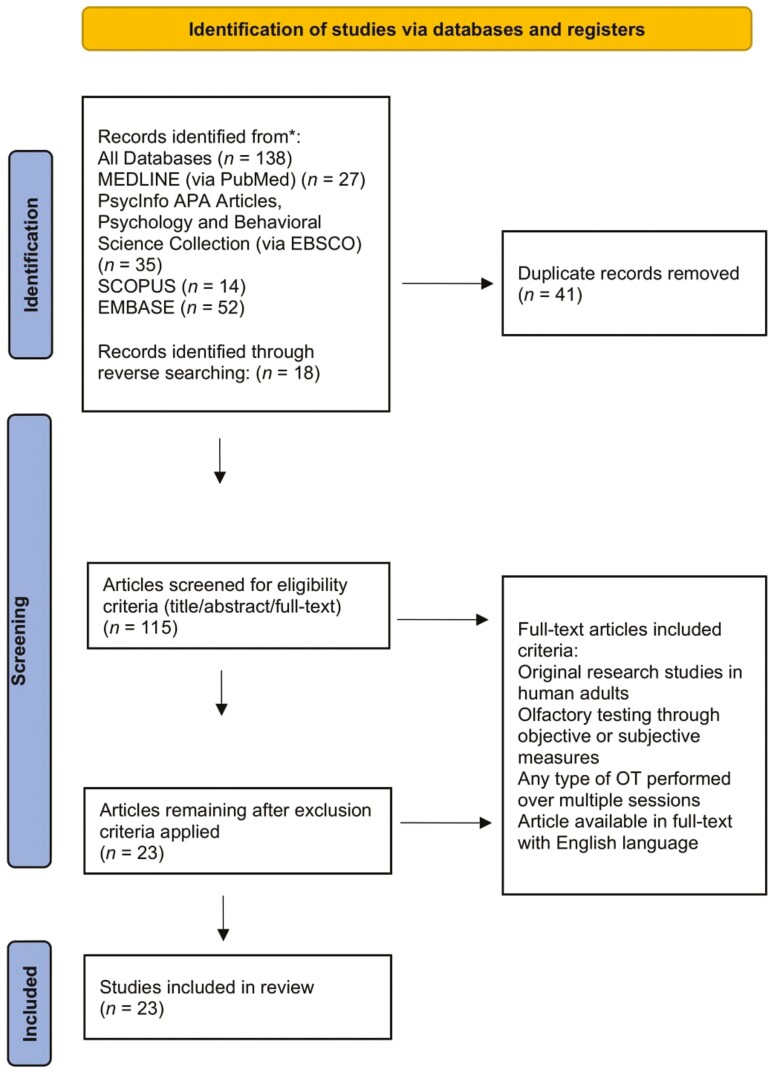
PRISMA diagram of screening method. OT = olfactory training; PRISMA = Preferred Reporting Items for Systematic Reviews and Meta-Analyses.

The scoping review framework was selected in order to achieve a broad understanding of OT to identify relevant data and possible gaps in the literature (Arksey & O’Malley, 2005). The framework presented by Arksey and O’Malley (2005) was used, adopting the five stages of conducting a scoping review. These are: (1) identifying research question, (2) identifying pertinent studies, (3) selecting studies to be used, (4) charting data collected, (5) summarizing and reporting results. The PRISMA-ScR (Preferred Reporting Items for Systematic Reviews and Meta-Analyses Extension for Scoping Reviews) was selected (see Figure 1).

#### Inclusion/exclusion criteria

The inclusion criteria were: (1) original research studies with human populations (no review articles included); (2) participants included adults aged 55 and older; (3) any type of OT performed; (4) a form of olfactory testing had to be performed. Additionally, the studies needed to be available in full text and in English. No limitations were made for gender, ethnicity, or cognitive status (i.e., mild cognitive impairment [MCI], dementia, healthy cognitive ability, etc.). If inclusionary criteria were not met (e.g., no OT or olfactory testing), the study was excluded.

#### Search strategy

A specialist librarian was consulted to help develop the search strategy, identify relevant databases, and create search terms. The databases were: PubMed (Medline), Embase, EbscoHost (APAPsycInfo, APA PsycArticles, Psychology and Behavioral Sciences Collection), and SCOPUS. A variety of search strings relevant to each key term (OT, aging, and cognitive impairment) were created for each database and included use of MeSH (Medical Subject Headings) terminology as appropriate. The literature search was conducted on April 5, 2023, and yielded an initial 138 results (see Figure 1). Backwards searching was conducted among the included studies to identify additional articles of relevance.

#### Screening and inclusion process

Two reviewers (M. Loughnane, R. Khalid Saifeldeen) independently reviewed the studies identified in the initial search. Each reviewer screened the article titles and abstracts to exclude duplicates. The reviewers met to discuss their findings from the initial screening and uncertainties were discussed and an agreement made. For the next round of screening, the two reviewers worked independently and assessed the remaining 115 results using abstract and full-text screening against the inclusion criteria. The reviewers compared findings and discussed discrepancies until consensus was reached. The screening resulted in the selection of 23 studies for inclusion in this review.

#### Data charting

The first reviewer (M. Loughnane) created an extraction table for the included articles, whereby key components were used to extract relevant data. The components included: study country, study aim, participant information, inclusionary/exclusionary criteria, study design, OT type and dosage, odors used (with concentrations), OT adherence measures, threshold testing, discrimination testing, identification testing, TDI composite used, cognitive and well-being measures, findings for OT and any other variables, and strengths and weaknesses of the study. Both reviewers extracted data from the identified studies for inclusion in the review (see Supplementary Table 1 for presentation of findings).

## Results

All studies included details regarding OT methodology, and all but two (Cha et al., 2022; Knudsen et al., 2015) reported the specific odorants used for OT. Cognitive variables were measured in seven studies, and at least one well-being factor was assessed in eight studies.

### OT Methodology

#### Odorants used for OT

A majority (*n =* 13) of the studies used the four COT odorants (citronellal, eugenol, eucalyptol, and PEA). Some (*n* = 5) compared groups using different types of odorants within the same OT procedure. Comparisons included use of the four standard COT odorants with multi-molecule fragrance mixtures (e.g., mandarin, sea odor, burnt rubber) or odors from household products (e.g., vanilla essence, natural toothpaste), or comparison between low-molecular-weight odors (e.g., cis-3-hexanol [cut grass]) and high-molecular-weight odors (e.g., gardocylene [woody/herbaceous]). Three studies changed the odors over the course of the OT intervention. For example, Altundag et al. (2015) compared the OT outcomes from a group of adults with postinfectious olfactory dysfunction (PIOD) who used the four classic single-molecule COT odorants for the entire 36-week intervention, whereas the modified OT group changed their four training odorants every 12 weeks and included odors from single molecule and mixtures of odorants. The aim of the modified OT was to stimulate a greater number and variety of olfactory receptors via changing the odorants used for training over the course of the intervention to measure potential differences in training outcomes. The participants in the modified OT group had better olfactory discrimination and identification scores than the COT group, but these differences did not reach clinical significance in changes of TDI scores.

#### Classical olfactory training

The COT approach was utilized by 10 of the studies (see Supplementary Table 1). Many of the studies extended the training period beyond 12 weeks ranging from 12 up to 56 weeks to investigate whether longer OT periods would produce further benefits. Among the studies that compared olfactory outcomes over extended durations of COT, the steepest rates of olfactory improvement occurred between 13 and 18 weeks with gains tapering off after that point. Fornazieri and colleagues (2020) reported better olfactory outcomes for adults with olfactory dysfunction in their COT group, when compared to the group that trained with odors from commercial products, where the improvements recorded after 3 months of OT were nearly equivalent to the outcomes after 6 months of training. Likewise, Konstantinidis et al. (2016) reported improved olfactory function for adults with PIOD across TDI, threshold, identification and discrimination after 16 weeks of COT, and the improvements were sustained within their follow-up period at 56 weeks. The group that conducted COT for the full 56 weeks had better results than those with the shorter regimen; however, both had significantly higher scores than the control group, and shorter duration COT was associated with potentially sustainable olfactory improvements. Another manipulation to the COT protocol was Damm et al.’s (2014) comparison of COT for 16 weeks with either high- or low-intensity odor concentrations, and then an additional 16 weeks of COT with the opposite condition. Among adults with PIOD, 26% of those who trained using high concentrations for the first 16 weeks had improved TDI scores as compared to only 15% of those participants who trained using low-concentration odors for the first 16 weeks.

### Olfactory Assessment and OT Outcomes

Overall findings indicated that OT was effective in improving olfactory function in older adults. The studies included either healthy older adults who were free from cognitive or olfactory impairment, adults with olfactory impairment of varying degrees and etiologies, and adults with cognitive impairment due to MCI, dementia, or Parkinson’s disease. The Sniffin’ Sticks test was the most popular assessment used to measure olfactory function. Of the three studies that did not measure olfactory function with Sniffin’ Sticks, two used the UPSIT (Fornazieri et al., 2020; Patel et al., 2017) and Cha et al. (2022) used an adapted version of the YSK olfactory function (YOF test), an olfactory function test using culturally familiar Korean odorants. Cha et al. (2022) used only the smell identification subtest of the YOF, as the researchers reported that persons with dementia struggled with the threshold and discrimination portions of the test.

#### Control groups and adherence

An issue in evaluating the effectiveness of the OT interventions was a lack of detail concerning adherence to the training regimen. A smell diary kept by the participants was the most common form of adherence measure. Typically, participants were instructed to evaluate their olfactory function in a weekly diary. Additionally, experimenters would call participants roughly every 4 weeks to ask about olfactory function and adherence with the OT procedure. Alternatively, some studies asked participants to provide ratings in their smell diaries after every training session, while many of the studies reported no adherence measures. Although adherence was measured among some studies, rarely was any adherence level reported and only a few of the studies reported dropout rates due to lack of adherence to the training protocol (e.g., Cha et al. (2022) reported dropout after a failure to participate in at least 20% of the sessions, which was easily measurable as the researchers administered the OT intervention). Fornazieri et al. (2020) measured adherence rates and OT efficacy among adults with varying types of olfactory loss. Researchers evaluated OT adherence at 3 and 6 months’ visits. Participants were asked about their training adherence, and they defined a lack of adherence as a participant not following the protocol or stopping training during the study. OT adherence was highest for the first 3 months at 88% of participants, and then significantly dropped off by 6 months where only 56% of participants were still adhering to the protocol. All of the participants who discontinued OT said they did so because they did not experience a noticeable improvement in their olfactory functioning.

#### Healthy older adults

Three studies investigated OT with healthy older adults (Oleszkiewicz., 2021; Schriever et al., 2014; Wegener et al., 2018). A summary of key findings from these studies can be found in Supplementary Table 1. All three of these studies reported improvements in multiple areas of olfactory function following the OT intervention. Whereas the olfactory outcomes from Schriever and colleagues (2014) and Wegener and colleagues (2018) arose from the classic form of OT (varying between 13 and 20 weeks in duration), Oleszkiewicz and colleagues (2021) investigated olfactory, emotional, and cognitive well-being among older adults utilizing either single-molecule odors or odor mixtures in their OT intervention (see Supplementary Table 1 for odor and OT summary). The researchers reported that odor threshold improved for the adults who used single-molecule odor stimuli in their OT intervention. Those adults in the single-molecule OT group and odor mixtures group experienced reduced cognitive decline compared to the control group. Additionally, the single-molecule group demonstrated significant improvements in Montreal Cognitive Assessment (MoCA; Nasreddine et al., 2005) scores. While potentially limited, these findings suggest that single-molecule OT stimuli were more effective for both olfactory and cognitive outcomes than odor-mixture OT stimuli among cognitively healthy older adults.

#### Adults with olfactory impairment

Fourteen of the studies included adults with olfactory impairment due to PIOD, post-traumatic olfactory dysfunction (PTOD), post-viral olfactory dysfunction (PVOD), or idiopathic etiologies (see Supplementary Table 1). Generally, OT was effective in improving olfactory function among adults with olfactory impairment as most frequently captured in improved TDI scores. Differentiating etiology of olfactory loss with specific outcomes showed that participants with PTOD or idiopathic olfactory dysfunction (OD) were associated with significantly lower odds of improvement for TDI scores (Liu et al., 2020). Adults with PIOD who had lower baseline olfactory performance were more likely to show improved identification and discrimination function, and Liu and colleagues (2021) also reported that clinically relevant improvements in threshold were less likely among those participants with increased age. Among the four studies who solely investigated participants with PIOD, three reported significantly improved TDI scores, and OT duration ranged from 16 to 56 weeks. Variability in the significance of OT outcomes was present for studies that compared the same type of olfactory loss among participants of similar age range and OT procedure (e.g., Geißler et al., 2014; Gellrich et al., 2018; see Supplementary Table 1). The main differences between the two studies were differences in sample size, training length (although the shorter protocol with the larger sample size resulted in measured improvements across all olfactory variables), and inclusion of a control group.

Poletti et al. (2017) explored the influence of molecular weight and olfactory improvement through OT for a group of adults with PTOD or PVOD. Participants performed OT for 5 months using either low-molecular-weight odors or high-molecular-weight odors. Contrary to their hypothesis, the low-molecular-weight odors did not lead to greater olfactory improvement over training with high-molecular-weight odors. In fact, participants in the high-molecular-weight training group had significantly improved thresholds for PEA (specifically among PVOD participants), whereas similar gains were not made in the low-molecular-weight (LWM) group.

#### Adults with cognitive impairment

Only Cha and colleagues (2022) examined OT and olfactory outcomes among persons with dementia. They provided an intensive OT method that lasted 15 days and included training twice a day using 40 odorants. This OT design was unique to the others in the large number of odorants, long session length (i.e., 15 min per session) and short duration of training period. The experimenters facilitated the OT sessions as opposed to the other studies that relied on self-administration, which also gave insight to OT adherence. The researchers measured olfactory function through a measure of identification only, as they reported the participants struggled to complete other olfactory tests (e.g., threshold detection). As compared to baseline performance, persons with dementia had significantly worse identification scores after OT, and these were worse than the control group who did not perform OT. The researchers did not distinguish between types of dementia among the participants, and the OT intervention was unique in short training period duration and large number of odorants. The two studies of MCI used Sniffin’ Sticks with all of the subtests, and only Haehner et al. (2022) reported any change with significantly improved discrimination after their 4-month training period (see Supplementary Table 1).

### Cognitive Assessment and Outcomes

Seven studies included cognitive measures in conjunction with OT. The key findings from the studies of OT and cognition in older adults are presented in Table 1. Cognitive screeners such as the MoCA and Mini-Mental State Examination (MMSE; Folstein et al., 1975) were used in all studies that explicitly measured cognition to provide a screening tool for MCI and dementia, and as a rudimentary measure of global cognitive function. The results were mixed for the impact of OT and improved global cognitive function, where only two studies reported improved MoCA or MMSE scores while the other five reported no change. In terms of comprehensive cognitive assessment, most studies included cognitive batteries assessing at least two cognitive domains, including memory, visuospatial function, language, verbal learning and memory, executive function, and attention. Results were mixed on the impact of OT across multiple cognitive domains among an older population either with olfactory or cognitive impairment, but six of the seven included studies reported significant improvement in at least one cognitive domain (see Table 1). Of the cognitive domains assessed, verbal fluency, specifically semantic verbal fluency, and working memory showed the most consistent improvement, whereas there were no reported significant improvements of executive function, visual memory, or naming. The length of OT and associated cognitive improvement varied from 15 to 20 weeks, suggesting that a relatively short- to medium-length course of OT could provide sufficient time to produce at least some degree of measurable benefit among older adults with and without cognitive impairment.

**Table 1.** Main Findings From Olfactory Training and Cognitive and Well-Being Outcomes

References	Cognitive assessment(s)	Well-being assessment(s)	Cognition outcomes	Well-being outcomes
Cha et al. (2022)	• CERAD-K • VFT (semantic verbal fluency) • K-BNT (naming) • MMSE-KC (global cognitive function) • WLMT (working memory) • CPT (visuospatial function) • WLRT (verbal memory encoding) • WLRcT (verbal memory retrieval) • CRT (visual memory) • SCWT (executive function)	• IADL-K • SGDS-K (depression scale)	+ Semantic verbal fluency + Naming + Working memory + Verbal memory encoding + Verbal memory retrieval No change: • Global cognitive function • Visuospatial function • Visual memory • Executive function	+ Significantly reduced depressive symptoms No change: • IADLs
Chen et al. (2022)	• MMSE (global cognitive function) • WST (vocabulary test/premorbid intelligence) • WMS-R (short-term memory) • CERAD (semantic verbal fluency) • BNT (naming) • NAI (Labyrinth test/planning ability) • TMT-B (executive function) • FLei (subjective impairment for cognitive complaints)	• BDI (depression scale)	+ Global cognitive function + Memory span backwards/working memory (control group only) No change: • Vocabulary test • Semantic verbal fluency • Naming • Executive function • Subjective impairment	No change: • Depressive symptoms
Haehner et al. (2022)	• MMSE (global cognitive function) • CERAD-NP (verbal and visual learning, visual memory delayed recall) • WMS-R (digit span forward—working memory) • TMT (trail making test—executive function) • BNT (naming) • NAI (age inventory) • FWT (Labyrinth test—executive function)	• BDI-II (depression scale)	+ Working memory No change: • Global cognitive function) • Verbal and visual learning • Visual memory • Executive function • Naming • Age inventory	No change: • Depressive symptoms
Knudsen et al. (2015)	• MMSE (global cognitive function) • RAVLT (verbal learning and memory) • BNT (naming)	• Hyposmia and QoL	PD-OT group - Naming - Verbal learning and memory No change: • Global cognitive function	+ PD patients moderately bothered by hyposmia + 52% reported improved olfactory function would positively affect their QoL
Oleszkiewicz et al. (2021)	• MoCA (global cognitive function) • AD8 (dementia screening interview, cognitive decline measure) • COWA (phonemic and semantic verbal fluency)	• BDI (depression scale) • PANAS (affect scale)	+ Global cognitive function (simple OT group) + Increased cognitive decline (control group) No change: • Verbal fluency	No change: • Depressive symptoms • Affect state (positive or negative)
Oleszkiewicz et al. (2022)	• MoCA (global cognitive function) • COWA (phonemic verbal fluency) • Semantic verbal fluency	• BDI (depression scale) • PANAS (affect scale)	+ Semantic verbal fluency (classic OT) No change: • Global cognitive function • Phonemic verbal fluency	No change: • Depressive symptoms • Affect state
Wegener et al. (2018)	• MoCA (global cognitive function) • COWA (phonemic and semantic verbal fluency) • AVLT, *Learninglist* (short-term memory) • Attention and Concentration test	• BDI-1 (depression scale) • WHO Well-being Index (QoL) • Cognitive age questionnaire	+ Semantic verbal fluency + Short-term memory subtest No change: • Global cognitive function • Phonemic verbal fluency • Attention and concentration	+ Reduced depressive symptoms No change: • QoL • Cognitive age, except perceived age subsection

*Notes*: AD8 = eight-item Informant Interview to Differentiate Aging and Dementia; AVLT = auditory verbal learning test; BDI = Beck Depression Inventory; BNT = Boston Naming Test; CERAD = Consortium to Establish a Registry for Alzheimer’s Disease Assessment Packet; CERAD-K = Consortium to Establish a Registry for Alzheimer’s Disease Assessment Packet—Korean Version; CERAD-NP = Consortium to Establish a Registry for Alzheimer’s Disease Neuro-Psychological; COWA = Controlled Oral Word Association Test; CPT = Cognitive Performance Test; CRT = Constructional Recall Test; FLei = Mental Ability Questionnaire; FWT = Five Word Test; IADL-K = Korean-Instrumental Activities of Daily Living; K-BNT = Boston Naming Test—Korean Version; MMSE = Mini-Mental State Examination; MMSE-KC = Mini-Mental State Examination—Korean Version; MoCA = Montreal Cognitive Assessment; NAI = Nurnberg Age Inventory; OT = olfactory training; PANAS = Positive and Negative Affect Schedule; PD = Parkinson’s disease; QoL = quality of life; RAVLT = Rey Auditory Verbal Learning Test; SCWT = Stroop Color and Word Test; SGDS-K = Geriatric Depression Scale-Short Form—Korean Version; TMT = Trail Making Test; TMT-B = Trail Making Test Part B; VFT = Verbal Fluency Test; WLMT = Word List Memory Test; WLRcT = Word List Recognition Test; WLRT = Word List Recall Test; WMS-R = Wechsler Memory Scale—Revised; WST = Wortschatztest (German Vocabulary Test).

#### Verbal fluency

Findings regarding verbal fluency were promising. Five studies included measures of verbal fluency, all of which assessed semantic verbal fluency and three assessed phonemic verbal fluency (see Table 1 and Supplementary Table 1). Of those studies, three reported improvements in semantic verbal fluency, whereas none found changes in phonemic fluency scores measured from baseline to post-OT periods. The improvements in semantic verbal fluency were found among healthy adults, persons with dementia, and adults with OD from varying etiologies. The study procedures largely followed a COT approach, except for Cha et al. (2022) who employed a COT framework but used 40 odorants instead of the typical four.

#### Memory

Five studies included some form of memory evaluation, and benefits from OT were seen most frequently in working memory; however, gains were also reported in short-term memory and verbal learning (see Table 1 and Supplementary Table 1). The three studies that measured working memory all reported significant improvements and included participants who were cognitively healthy, persons with dementia, and adults with MCI. Among the adults with MCI, improved working memory was only recorded in the control group, where adults with MCI performed a placebo OT protocol, utilizing odorless stimuli jars (Chen et al., 2022). No significant improvements were reported for visual memory.

### Well-Being Assessment and Outcomes

Well-being measures such as depression scales (most commonly the Beck Depression Inventory-1; Beck et al., 1988) and QoL questionnaires were included in eight of the studies (see Table 1). Of the six studies that measured depression, two reported significant reductions in depressive symptoms among both cognitively healthy and persons with dementia following OT (Cha et al., 2022; Wegener et al., 2018). It is worth noting the difference in the OT protocol between the two studies that produced significant reduction in depressive symptoms. Wegener and colleagues (2018) employed a traditional COT approach among cognitively healthy older adults, whereas Cha et al. (2022) used an intensive OT intervention for persons with dementia that lasted 15 days and involved participants smelling 40 odorants twice a day.

## Discussion and Implications

The results of the scoping review suggest that OT provides multiple benefits to some older adults, including those with cognitive impairment. However, the extent of the literature examining OT with older adults is limited and should be cautiously interpreted, as OT is not a cure or treatment capable of restoring complete olfactory function. Olfactory perception declines with increasing age, and is exacerbated by age-related neurodegenerative diseases such as AD (Doty et al., 2017; Hedner et al., 2010; Hummel et al., 1997; Olofsson et al., 2020). The findings provide preliminary evidence to suggest that benefits from OT may extend beyond measurable changes in olfactory function to include improved cognitive function and amelioration of depressive symptoms (Cha et al., 2022; Chen et al., 2022; Wegener et al., 2018). There is potential to adopt a simple, nonpharmacological, home-based intervention that can be self-administered with relatively minimal risk. The potential benefit from promoting the adoption of such an intervention is substantial, to support well-being as part of normal aging to help sharpen olfactory acuity and to provide protection from cognitive decline.

The findings demonstrate that simple COT produces some significant outcomes for older adults (e.g., Hummel et al., 2009; Konstantinidis et al., 2016; Wegener et al., 2018). Continued research and refinement of the OT protocols may serve to maximize the potential benefit, especially in differentiating training length, intensity, and stimuli to maximally benefit the needs of the individual, whether that be olfactory outcomes for someone experiencing hyposmia, olfactory loss due to upper respiratory tract infections, or a person with dementia who would benefit not only from improved olfactory outcomes but the associated cognitive and well-being benefits.

Within verbal fluency, semantic fluency was sensitive to change over the course of OT, while phonemic fluency was not. More compelling was that improved semantic verbal fluency was reported among not only healthy adults but also adults with OD and persons with dementia. Interestingly, verbal fluency is believed to remain intact in the normal aging process, whereas both semantic and phonemic fluency are impaired in neurodegenerative diseases such as AD and MCI (Fama et al., 2000; McDonnell et al., 2020; Nutter-Upham et al., 2008; Rinehardt et al., 2014). Furthermore, semantic verbal fluency performance is more sensitive to AD and can serve as a screening tool for detecting early-stage dementia and MCI (Brickman et al., 2005; McDonnell et al., 2020; Pakhomov et al., 2018). Verbal fluency draws from both executive function and language processing (Allen & Fong, 2008; Ruff et al., 1997; Whiteside et al., 2016) and the cortical areas activated by odor identification overlap with established cognitive correlates associated with semantic verbal fluency (Abrahams et al., 2003; Riello et al., 2021). Therefore, it is possible that sensory stimulation provided through a period of OT could provide adequate activation in these shared regions not only for measurable change in odor identification but semantic verbal fluency as well; however, this interpretation requires further exploration.

The utility of OT as a means of preventing or ameliorating cognitive decline, while promising, has yet to be established. Only a single study (Oleszkiewicz et al., 2021) investigated the impact of an OT intervention on cognitive decline among normally aging adults without olfactory impairment. The results of their OT intervention provided insulation against cognitive decline, as the control participants who did not perform OT showed declined performance on the MoCA.

It is worth considering the influence of ceiling effects on these well-being measures in terms of outcomes associated with OT. For the included studies that measured depression, most reported no change in symptom level following OT (see Table 1). However, two did report significant reduction in depressive symptoms (Cha et al., 2022; Wegener et al., 2018) and another reported significantly improved QoL (Yilmaz et al., 2022), suggesting that there could be a potential connection and benefit. Four of the six included studies that measured depression, excluded participants based on current or history of psychiatric illness or elevated depressive symptoms. The lack of results may be due in part to ceiling effects, and while including depression measures is highly relevant among an aging population, especially among those with dementia, exploring the benefit of OT for a condition such as depression may benefit from more in-depth analysis where researchers account for varying levels of depressive symptoms among the participants and account for baseline ceiling effects.

### Future Research

Further research is needed across specific participant groups as it remains unclear whether a generic approach to OT is the best way forward. There may be manipulations and alterations needed in the OT protocol, related to the duration of the intervention, and more nuanced consideration for the composition of the specific odors used for training (e.g., single-molecule odors, multi-molecule odor mixtures, low vs high concentration, etc.). This may lead to differentiated outcomes across olfaction and cognition more relevant for specific populations (e.g., young adults recovering from coronavirus disease-related anosmia vs persons with dementia with olfactory dysfunction).

There is potential to adapt an OT protocol to maximize benefit to a variety of populations. These findings establish a relationship between OT and semantic verbal fluency (Cha et al., 2022; Oleszkiewicz et al., 2022; Wegener et al., 2018). Therefore, an OT intervention may benefit someone experiencing frontotemporal dementia or primary progressive aphasia, where issues in language, specifically verbal fluency, present early and significantly (Libon et al., 2009; Rohrer & Rosen, 2013; Seelaar et al., 2011; van den Berg et al., 2022). Future research should consider differentiated types of dementia in investigating the outcomes from OT so that protocols could be developed to maximally align the benefits to individual patients.

Future research might also create multimodal, multisensory, cognitively engaging OT protocols to potentially enhance overall outcomes. Olofsson and colleagues (2021) recently called for the evolution of standard or COT methodology to include a cognitive training element, such as a training game using odors, or utilization of computerized OT. These advances could facilitate elicitation of more precise data on how participants engage with odors, their adherence to the training protocol, as well as opportunities to manipulate the intensity of the cognitive component of training through adjusted difficulty levels based on performance feedback. A reliable measure of adherence to the training protocol was a persistent issue across the OT protocols. Cha et al. (2022) reported strong adherence data as the researchers facilitated the smell training protocol with the participants, whereas other protocols relied on self-reported adherence data, which may be subject to overestimated adherence behavior (Stirratt et al., 2015). More advanced and reliable adherence data may help to establish the accurate impact the OT protocol has on treatment outcomes and their effect sizes.

Given the association between olfactory impairment and long-term mortality, more research is needed to better understand this relationship as this elevated risk is only partly explained by neurodegenerative disease and weight loss, and remains independent of other common confounds (Liu et al., 2019). What remains less clear is the potential for OT to mitigate any of this risk. Longitudinal follow-ups in studies with OT may provide an opportunity to gain insight into the relationship between OT and improved outcomes and variables such as mortality, as well as evaluating any potential training necessary for maintenance of the outcomes.

### Limitations

A limitation of this review was not discussing results regarding brain imaging data of OT among an older population. In a systematic review, Vance et al. (2023) explored whether OT improved cognition and altered brain structure and connectivity, including studies that addressed either cognitive outcomes or neuronal imaging. Our review identified only one neuroimaging study, which was not described by Vance et al. (2023): Chen et al. (2022) collected BOLD response data to their OT intervention among adults with MCI and observed an increase in BOLD response in the orbital-frontal cortex and frontal gyrus in response to an odor. Additionally, they found a positive correlation between changes in TDI scores and BOLD responses in these frontal areas, which further supports the findings previously discussed. In total, we identified three studies that employed neuroimaging among adults aged 55 and older (i.e., Chen et al., 2022; Gellrich et al., 2018; Pellegrino et al., 2019). Given the small sample size of neuroimaging studies conducted with adults aged 55 and older, it is difficult to reach any consensus in the results as many more studies will need to be conducted.

Additionally, this review only included studies that were available in full text in the English language. This may have contributed toward the bias of studies originating in European countries. The majority of the reviewed studies were conducted with participants based in Germany (*n* = 16), with a total of *n* = 20 studies involving European populations. The other countries represented were Brazil, South Korea, and the United States. While findings regarding olfactory function outcomes may be generalizable, interpreting specific changes, for example, in the threshold for specific odors trained, or use of odors available from commercial products available in grocery stores (Fornazieri lens, 2020), may need to be interpreted in the context of the participant culture and region.

## Conclusion

This review of the literature concerning OT and older people has demonstrated the potential utility of OT as a means of improving olfactory function as well as cognition and well-being with varied effect on adults both with and without cognitive impairment. Duplication of these studies is needed, especially in controlled studies, with more comprehensive neurocognitive assessments, in order to more clearly investigate the relationship between olfaction, OT, and cognition with age.

## Supplementary Material

igae044_suppl_Supplementary_Table

## Data Availability

Additional information on methods or materials supporting this review are available upon request. No primary data were collected for this study. To our knowledge, no studies included in this review were preregistered.
