# Supplementary material for: Aging and Olfactory Training: A Scoping Review
Source: Innov Aging. 2024 May 30;8(6):igae044. doi: 10.1093/geroni/igae044 (PMC11176978; doi:10.1093/geroni/igae044)
Supplement: igae044_suppl_Supplementary_Table [file igae044_suppl_supplementary_table.docx]

***Innovation in Aging* Supplementary Material: Loughnane et al. Ageing and Olfactory Training: A Scoping Review.**

**Supplementary Table 1. Summary of OT and Significant Findings**

| **References and Country** | **Participants and Sample Size** | **OT Dosage** | **OT Odours** | **Olfactory test** | **Olfactory Outcome** | **Cognitive and Well-being Outcomes** |
| --- | --- | --- | --- | --- | --- | --- |
| Altundag et al. (2015), Turkey | Adults with PIOD  *N* = 85  (45.6years ± 10.5)  Groups: MOT, COT, control (no OT) | 36 weeks  2x/day  4 odourants  10s smelling/odour  10s break between odours  5min odour exposure/ session | -Citronella, eucalyptol, eugenol, and phenyl ethyl alcohol (PEA) (36 weeks for COT; 12 weeks for MOT)  - Jasmine, tangerine, thyme, and menthol (next 12 weeks for MOT)  - Gardenia, rosemary, bergamot, and green tea (next 12 weeks for MOT) | SS  VAS (1-10) | + D  + I  In MOT and COT groups  Rate of improvement tapered off after 24 weeks | n/a |
| Cha et al. (2022), South Korea | Persons with dementia  *N* = 65 (m= 85years, range = 65-97)  Groups: IOT, control (no OT) | 2 weeks (15d)  2x/day  30 total sessions  40 odourants/session  15 min/session | Odours not reported  Odours reported as low intensity concentration | YSK, odour identification subtest only | -I (sig. worse for IOT group) | + Semantic verbal fluency  + Naming  + Working memory  + Verbal memory encoding  + Verbal memory retrieval  + Sig. reduction in depression score |
| Chen et al. (2022), Germany | Adults with MCI  *N* = 38  Groups: OT group (72.7years ± 4.9), control (odourless OT) (70.6years ± 7.0) | 16 weeks  2x/day  2x/session  4 odourants/session  15s smelling/odour | Citronellal, eugenol, eucalyptol, and PEA | SS | No change: T, D, I, TDI | + Working memory |
| Damm et al. (2014), Germany | Adults with PIOD  *N* = 171 (54.6years ± 9.6),  Groups: high intensity OT first, low intensity OT first | 32 weeks (16 weeks using high concentration odours and 16 weeks using low concentration odours)  2x/day  2x/session  4 odourants/session  15s smelling/odour | Citronellal, eugenol, eucalyptol, and PEA | SS | + TDI  + Improvements in olfactory function higher in high concentration training group after 18 weeks | n/a |
| Fleiner et al. (2012), Germany | Adults with OD from varying aetiologies  *N* = 46 (59.17years ± 13.25)  Groups: OT only, OT and steroid treatment | 34 weeks (8 months)  2x/day  4 odourants/session  10s smelling/odour | Rose, orange, citrus, peppermint, raspberry, chocolate, vanilla, cinnamon, leather | SS | + TDI (whole group)  + I (whole group) | n/a |
| Fornazieri et al. (2020), Brazil | Adults with OL from varying aetiologies  *N* = 25 (range 22-82years)  Groups: classic OT, modified OT (training odours from commercial products) | 26 weeks (6 months)  2x/day  4 odourants  10s smelling/odour  Classic OT: odours replaced at 3 months  Modified OT: instructed to change products every week | Classic OT group:  PEA, eugenol, citronellal, and eucalyptol.  Modified OT group: coffee powder (2 tbsp), vanilla essence (Dr. Oetker), cloves (10), toothpaste (Colgate Natural, 20 g), and mandarin juice (Maguary, 20 mL) | UPSIT | + Improved olfactory function. Improvement marginally better in Classic OT group   - 3mo of training nearly as effective as 6mo, and adherence rates highest at 3 months (88%) | n/a |
| Geißler et al. (2014), Germany | Adults with UTRI OL  *N* = 39 (56years ± 8) | 32 weeks  2x/day  4 odourants  10-20s smelling/odour  Smelled in random order | Citronellal, eugenol, eucalyptol, and PEA | SS | + TDI  + D | n/a |
| Gellrich et al. (2018), Germany, Austria & Switzerland | Adults with UTRI OL  *N* = 61 (range = 45-80years)  Groups: healthy controls (no OT), adults with hyposmia | 12 weeks  2x/day  4 odourants  10s smelling/odour with focused attention on odour | Citronellal, eugenol, eucalyptol, and PEA | SS | + TDI (53% had improvement at clinically sig. level)  + T  + D  + I | n/a |
| Haehner et al. (2013), Germany | Adults with PD  *N* = 70 (range = 43-73years)  Groups: OT, control (no OT) | 12 weeks  2x/day  4 odourants  10s smelling/odour with focused attention on odour | Citronellal, eugenol, eucalyptol, and PEA | SS | + TDI  + D  + I  + T, specifically for OT odourants | + Working memory |
| Haehner et al. (2022), Germany | Adults with MCI  *N* = 37 (70.9years ± 6.2)  Groups: OT, and placebo (odourless OT) | 17 weeks (4 months)  2x/day  4 odourants  20s smelling/odour | Citronellal, eugenol, eucalyptol, and PEA | SS | + D | n/a |
| Hummel et al. (2009), Germany | Adults with OD from varying aetiologies  *N* = 56 (57.8years ± 12)  Groups: OT, control (no OT) | 12 weeks  2x/day  4 odourants  10s smelling/odour | Citronellal, eugenol, eucalyptol, and PEA | SS | + TDI (28% had improvement at clinically sig. level)  + T, for citronellal, eugenol, and PEA | n/a |
| Knudsen et al. (2015), Denmark | Adults with PD  *N* = 80  Groups: PD-OT (64.2years ± 8.2) , PD- no OT (67years ± 7.3), healthy adults-OT (62.7years ± 8.9) | Odour Identification Training  1 day  2 sessions  10 min/session  10-minute break  16 odours presented in random order  Verbal and visual descriptions presented alongside each odour | Odours not reported | SS | + D (OT groups)  + I (OT groups)  TDI and threshold not reported | PD-OT group  - Naming  - Verbal learning and memory  + PD patients moderately bothered by hyposmia  + 52% reported improved olfactory function would positively affect their QoL |
| Konstantinidis et al. (2016), Greece | Adults with PIOD  *N* = 111  (62.9years ± 6.2)  Groups: OT short-term, OT long-term, control (no OT) | 16 weeks OR 56 weeks  2x/day  4 odourants  10s smelling/odour  10s break between odours  5min odour exposure/session | Citronellal, eugenol, eucalyptol, and PEA | SS  VAS (1-10) | + TDI  + T  + D  + I  + VAS rating   - Steepest curves of improvement from baseline to 16 weeks - 16 weeks of OT provided sustainable results lasting at least 56 weeks | n/a |
| Liu et al. (2020), Germany | Adults with OD  *N* = 601  (57years ± 11)  Groups: OT, control (no OT) | 30.6 weeks (M duration ± 8.4 weeks)  2x/day  4 odourants  10s smelling/odour  10s break between odours  5min odour exposure/session | Citronellal, eugenol, eucalyptol, and PEA | SS | + TDI   - Improvement less likely for higher baseline TDI and older age - PIOD more likely to improve   T, I, and D not reported | n/a |
| Liu et al. (2021), Germany | Adults with PIOD  *N* = 153 (58.7years ± 7.3)  Groups: OT short-term, OT long-term, control (no OT) | 25.8 weeks (M duration ± 8 weeks)  2x/day  4 odourants  15s smelling/odour | Participants received either:   - Single molecule odours for duration (Citronellal, eugenol, eucalyptol, and anethol) - Multi-molecule odours for duration (PEA, eucalyptol, citronellal, eugenol) - 12 multi-molecule odours alternated in groups of 4 every 8 weeks (PEA, eucalyptol, citronellal, eugenol, cinnamon, thyme, chocolate, peach, coffee, lavender, honey, strawberry) | SS | - Improvements in D and I more likely in those with lower baseline function - Clinically relevant T improvements less likely for those older in age   Outcomes regarding odours trained and olfactory function not reported | n/a |
| Oleszkiewicz et al. (2021), Germany | Healthy adults  *N* = 68 (62.8 years ± 8.9)  Groups: single molecule odours OT, odour mixtures OT, control (no OT) | 16 weeks (M = 4.1 months ± 0.42)  2x/day  9 odourants  20s smelling/odour  • Odours aimed to provide trigeminal stimulation and evoke memories and associations (e.g., sea breeze, freshly mown hay) • Odours selected experts to likely evoke pleasant memory, positive associations, and similar intensities | Single-molecule Odours: • Eugenol, eucalyptol, citral, menthol, anethol, ethylvanillin, cumarine, calone, butanol  Odour mixtures (essential oils) • Clove bud, eucalyptus, yellow mandarin blend, peppermint, star anise, vanilla mauvais, tonka beans absolute, sea odour, burnt rubber | SS  VAS (0-100)  Odour intensity rating (0-11) | + T (single molecule OT) | + Global cognitive function, simple OT (single molecule OT group)  + Increased cognitive decline (control group) |
| Oleszkiewicz et al. (2022), Germany | Adults with OD from varying aetiologies  *N* = 55 (58.2 years ± 11.3)  Groups: classic OT, intense OT | 15 weeks (M= 208.6 days ± 64.3, range= 108-340 days)  Classic OT= 2x/day, intense OT= 4x/day  5 odourants  30s smelling/odour  Session intervals at either 12 or 6 hours  Odours changed at 3 months OT | 0-3 months:  • Grapefruit, lavender, lemon grass, ylang-ylang, peppermint  3-6 months:  • Menthol, thyme, tangerine, green tea, bergamot | SS  Retro-nasal olfaction  IOQ | + T (classic OT)  + D  + I | + Semantic verbal fluency (classic OT) |
| Patel et al. (2017), USA | Adults with PIOD or idiopathic OL.  *N* = 35 (range = 39-71years)  Groups: OT, control (no OT) | 26 weeks (6 months)  2x/day  4 odourants  15s smelling/odour  15s break between Odours  Instructed to focus on remembering what the doors smelled like prior to anosmia | Rose, lemon, eucalyptus, clove (essential oils)  Participants obtained essential oils themselves, brand and concentration unspecified | UPSIT | + 32% of OT participants sig. improved olfactory function  + 13% of control participants sig. improved olfactory function   - Use of random concentration of essential oils for OT potentially comparable in olfactory outcomes to controlled concentrations | n/a |
| Pellegrino et al. (2019), Germany | Adults with PIOD  *N* = 42 (52.2 years ± 12.1)  Groups: OT-anosmic, OT- hyposmic | 24 weeks (M = 7.1 months ± 1.4)  2x/day  4 odourants  15s smelling/odour  Odour stimuli replaced at 12 weeks | Citronellal, eugenol, eucalyptol, and PEA | SS | + TDI (overall improvement, sig. improvement for OT-anosmia group)  + T (overall improvement, sig. improvement for OT-anosmia group)  + I (non sig. level) | n/a |
| Poletti et al. (2017), Germany | Adults with PTOD or PVOD  *N* = 96 (59.4 years ± 12.6)  Groups:  OT-LWM, OT-HWM | 21 weeks (5 months)  4x/day  4 odourants  10s smelling/odour | LMW:  Cis-3-hexanol, 140.12g/mol (cut grass), ethyl maltol, 140.14g/mol (caramelised sugar), pinene alpha, 136.23g/mol (essential oil), ocimene, 136.23g/mol (citrus)  HWM:  Fructone, 174.19g/mol (green apple), ehtyl vanilline, 166.18g/mol (vanilla), gardocylene, 220.21g/mol (woody/herbaceous), irone alpha, 206.32g/mol (floral/fruity) | SS  Intensity rating (0-10, no smell to max. intensity) | + TDI (overall improvement, sig. improvement for PVOL)  + T, for PEA (HWM for PVOL)   - No sig. difference in overall improvement between LWM (36% improved) vs. HWM (38% improved) - Improvement better for PVOD than PTOD | n/a |
| Schriever et al. (2014), Germany | Healthy adults  *N* = 91 (81years ± 8.6)  Groups:  OT, control (no OT) | 13 weeks (3 months)  2x/day  4 odourants  30s smelling/odour | Citronellal, eugenol, eucalyptol, and PEA | SS   - Odour detection not measured | + T (non. sig.)  + I (non. sig.)  -Olfactory function in control group   - OT may prevent olfactory deterioration with age | n/a |
| Wegener et al. (2018), Germany | Healthy adults  *N* = 91 (61.1years ± 8.7)  Groups: OT, control (Sudoku task) | 20 weeks  2x/day  4 odourants/session | Citronellal, eugenol, eucalyptol, and PEA | SS  IOQ | + T  + D  + TDI | + Semantic verbal fluency  + Short-term memory subtest  + Sig. reduction in depression score |
| Yilmaz et al. (2022), Turkey | Adults and children with drug-resistant epilepsy  *N* = 24 (20.8years ± 12.6, range = 9-65years)  Groups:  adult, paediatric | 13 weeks (3 months)  2x/day  1 odourant  30-40s smelling/odour  10-15s smelling/ nostril  10-15s smelling in front of septum for both nostrils | Lavandula Angustifolia | SS | + TDI (sig. improved in adult group)  + Trended improvement of T, D, I  - Reduced hyposmia rate (adult group) | + QoL |
| *Note*: COT=classical odour training; D=odour discrimination; HWM=high molecular weight; I=odour identification; IOT=intensive olfactory training; IOQ=the Importance of Olfaction Questionnaire; LWM=light molecular weight; MCI=mild cognitive impairment; MOT=modified odour training; OD=olfactory dysfunction; OL=olfactory loss; OT=olfactory training; PD=Parkinson’s disease; PEA=phenyl ethyl alcohol; PIOD=post-infectious olfactory dysfunction/loss; PIOD=post-infectious olfactory dysfunction/loss; PTOD=post-traumatic olfactory dysfunction/loss; SS=Sniffin’ Sticks; T=odour detection threshold; TDI=the sum of measures from Sniffin’ Sticks odour detection threshold, odour discrimination, and odour identification; UPSIT=University of Pennsylvania Smell Identification Test; UTRI=upper respiratory tract infection; VAS=visual analogue scale; YSK=olfactory function test.  Additional References:  Fleiner, F., Lau, L., & Göktas, Ö. (2012). Active olfactory training for the treatment of smelling disorders. *Ear, Nose & Throat Journal*, *91*(5), 198-215. <https://doi.org/10.1177/014556131209100508> | | | | | | |
